# Oral anaerobe bacteria—a common risk for cardiovascular disease and mortality and some forms of cancer?

**DOI:** 10.3389/froh.2024.1348946

**Published:** 2024-04-24

**Authors:** Anne Lise Lund Håheim

**Affiliations:** Dental Faculty, Institute of Oral Biology, University of Oslo, Oslo, Norway

**Keywords:** oral microbiota, anaerobe bacteria, antibodies, cardiovascular disease, cancer, chronic periodontitis, review

## Abstract

This review explores the results of research on oral health concerning cardiovascular diseases and some forms of cancer and is based on results from published systematic reviews and some studies. The research results will have a strong focus on exploring the relationship between different aspects of oral infections. The relationship between oral health parameters, cardiovascular diseases (CVD), and certain cancers was examined from different angles, including prospective analyses, in a population-based health study in Oslo from the year 2000 (Oslo II study). A major finding was that low levels of antibodies to the oral anaerobe *Tannerella forsythia* predict both CVD mortality in men with a history of myocardial infarction and incidence of bladder cancer in a random sample of men in the study. Low levels of antibodies to *Treponea denticola* predict the incidence of bladder and colon cancer in a random sample of men in the study. Both anaerobe bacteria are part of the so-called red complex of bacteria in chronic periodontitis together with *Pophyromonas gingivalis*. These three bacteria have different properties and are causal in chronic periodontitis. They migrate into the local tissues by adhering to the oral epithelium, break down soft and hard tissues, and spread via the circulation to organs distant from the mouth. This paper will give an overview of which oral health measures have been explored and associated with different CVD and cancer diagnoses and what scientific literature supports or contravenes our hypothesis. The oral microbiome is described with the most relevant bacteria related to microbiology, serum, autopsies, and associated causes such as alcohol. There will be a mention of the possibilities and limitations of different study designs. There seems to be a causal relationship between oral anaerobe bacteria and systemic diseases regulated by the immune system. This is seen alongside other well-known risk factors, especially for CVD. The prospective finding of a relation to the incidence of certain cancers and CVD is particularly intriguing. However, further research is needed to determine the biological mechanisms underpinning these associations.

## Oral health and systemic effects

### Background

Many observations and intervention studies confirm that oral health is related to systemic diseases and vice versa (1–3). This review is based on systematic reviews and a selection of primary studies investigating the correlation between oral health and different disease outcomes, particularly within the fields of cardiovascular diseases (CVD) and cancers (1). Systematic reviews have been carried out summarizing the results of several studies to gain the strength and precision of the risk estimates. Different search strategies were used for the book *Oral Infections and Systematic Diseases. Scientific Evidence from an Epidemiologic Perspective* (Table 1) (1).

**Table 1:** Search strategies for systematic reviews on oral infections and cardiovascular diseases and cancer.

• Cancer AND meta-analysis AND periodontal disease AND periodontitis AND review. • Myocardial infarction AND coronary heart disease AND meta-analysis AND review. • Stroke AND oral infection AND cohort study AND epidemiology NOT COVID-19. • Cardiovascular disease AND oral infection AND epidemiology NOT COVID-19 not cancer. • Mortality AND oral infection AND cohort study AND epidemiology NOT therapy NOT COVID-19.

Oral health can be assessed through various measures related to infections, such as gingivitis, chronic periodontitis, tooth extractions, other oral infections, or microbiological examinations. The measurements may be self-reported status, clinical measurements, laboratory measurements, or X-rays. The systemic diseases covered in this review are cardiovascular diseases including myocardial infarction (MI), ischemic heart disease, stroke, peripheral arterial disease, infective endocarditis, and different forms of cancer. The following diseases have also been studied, such as diabetes mellitus, lung diseases, rheumatoid arthritis, pregnancy and preterm births, Alzheimer's disease, gastric ulcers, infections of the face and neck, and total mortality. Different systemic diseases or conditions also have an impact on oral health. Hence, this diverse field requires an overview to summarize the current evidence to provide directions for further research.

### Oral health

Bacteremia can occur after tooth brushing, dental procedures, or dental infections (3). Oral infections, such as gingivitis, chronic periodontitis, and dental caries, may cause bacteremia, which later is identified by the presence of anaerobe bacterial DNA in the serum (3). Modern technology has given us much new information in microbiology in particular, but more research is needed to help us understand the biological mechanisms. Among the rich oral microbiota, anaerobe bacteria can break down the gingiva, periodontal tissue, and alveolar bone, and during caries development, employing various immunological and microbiological mechanisms (1–3). Intervention by treatment of chronic periodontitis has been shown to reduce CVD risk factors such as elevated C-reactive protein (hs-CRP) and interleukin (IL)-6 (4). The authors concluded that “severe generalised periodontitis causes systemic inflammation” and that “This is consistent with a causative role of periodontitis in atherogenesis.”

### Observed association between cardiovascular disease and cancer

Psaty and Vasan discussed the potential mechanisms for the finding of an association between myocardial infarction and later cancer incidence (5). Based on the study by Lemming et al. of the Rotterdam study, they raise the question “What is the explanation of the association between ST-segment elevation myocardial infarction with cancer incidence?”

This study and other epidemiologic studies, such as the Tromsø study in Norway and the Surveillance, Epidemiology, and End Results (SEER) registry and Medicare claims in the USA, also reported a high incidence of cancer after a myocardial infarction (5). In the Dutch study, there were no differences between the two groups concerning known risk factors for CVD, such as smoking, lipids, diabetes, obesity, and hypertension. However, other factors indicating infection or at least inflammation, such as CRP, hemoglobin level, platelet count, and leucocyte count differed. The added observation of hs-CRP levels being associated with ST-segment elevation myocardial infarction (STEMI) underscores the association with bacterial infections (5). This association between CVD and later incidence of cancer was seen by the authors as possible due to surveillance bias, occult cancer, or shared risk factors.

### Immunologic response to oral anaerobe bacteria

The spread of bacteria into the body triggers an immunological response leading to the development of antibodies. This process varies between individuals. Oral infections are common to a varying degree in all populations and the development varies a great deal among individuals (6, 7). The systemic effect can be assessed by the antibody response to oral anaerobe bacteria in prospective cohort analyses, such as in the Oslo II study from the year 2000 (8, 9). The oral cavity is rich in bacteria as over 700 different bacteria have been identified. These anaerobe bacteria have previously been categorized and the main category in chronic periodontitis is the so-called red complex, which consists of the bacteria *Tannerella forsythia* (TF), *Treponema denticola* (TD), and *Porphyromonas gingivalis* (PG) (10, 11). All infections cause an antibody response but to a varying degree among individuals. The results from the Oslo II study (increased risk of antibody levels of first quartile vs. fourth quartile) show TF predicts CVD mortality in men with a history of MI compared to MI-free individuals in a 12.5-year follow-up with a hazard ratio (HR) of 1.82 [95% confidence interval (CI) 1.12–2.94] (8). Further, in a 17.5-year follow-up of a random sample of men, TF predicted bladder cancer with an HR of 1.71 (95% CI 1.12–2.61) and TD colon and bladder cancers with an HR of 1.52 (95% CI 1.06–2.19) and an HR of 1.60 (95% CI 1.05–2.43), respectively (9). The prediction of low levels of antibodies to specific oral anaerobe bacteria assists in identifying individuals at risk for both MI and certain types of cancer. Oral bacteria have been identified in cardiovascular tissues, such as heart valves, atheromas, aneurysms, and pathological specimens of several different cancers (12–14). Without this knowledge, the observed association between MI before a diagnosis of cancer is difficult to explain (5).

These findings on antibodies shed light on the generalizability of our infection hypothesis of low levels of antibodies in infected individuals before CVD mortality and cancer incidence referred to by Psaty and Vasan (5).

### The aim of this review

This paper will give an overview of the oral health measures that have been explored and associated with CVD and certain cancers and what scientific literature supports or contravenes our hypothesis (1). The discussion will emphasize the potential and limitations inherent in different study designs. The results are related to microbiologic mechanisms, serum analyses, autopsies of CVD tissue, and cancer specimens. The influence of alcohol on oral infections will be explored (15).

## The opportunistic oral microbiome

The oral microbiota is rich and complex and contains many hundreds of different bacteria as well as yeasts, viruses, mycoplasmas, and protozoans. Within the oral cavity, there exists a multiorganism environment, the oral microbiota, that normally exists in harmony with the host. Thanks to the development of new technology, such as the pyrosequencing of 16S rDNA of bacteria, the number of identified microorganisms has increased greatly, with more than 700 species being identified in a previous report (16). This is a substantial increase compared to earlier times when only bacteria that were cultivated in laboratories were studied, limiting the understanding of the consequences of oral infections.

Socransky and colleagues studied the oral bacteria closely and were able to categorize them according to their properties and grouped them accordingly (3, 11). The groups were based on the stages of gingival and periodontal disease development, reflecting their metabolic status. The groups were named yellow, orange, green, purple, and red complexes, relating to different stages of oral infections and their ability to survive in increasingly anaerobe environments in periodontal disease and tooth pulp infections resulting from the development of caries.

Of particular interest in terms of risk for CVD and cancer is the red complex, which is composed of three anaerobe bacteria that have properties that support their function in tissue destruction in chronic periodontitis, namely *Tannerella forsythia* (TF), *Treponema denticola* (TD), and *Porphyromons gingivalis* (PG). The unifying features of these bacteria are that they are anaerobic, have extracellular proteolytic activity, perform complex anaerobic fermentation of amino acids, produce toxic metabolites, and have outer membrane vesicles. Through these attributes, the bacteria can survive in chronic periodontal infections, destroy periodontal tissue, and gain access to the blood circulation. The ability to destroy tissue explains the observations of bacteria and their bacterial DNA in serum, arterial walls, and tumor tissue. The biological mechanisms are not fully known. Further insight might provide testing opportunities to identify individuals at increased risk of these diseases.

The bacteria constituting the red complex are currently of greatest interest in CVD and cancer, even if bacteria such as *Fusobacterium nucleatum*, *Neisseria*, *Streptococcer*, *Veilonella*, and others have been identified in the body. The polymicrobial approach has been important in giving a better understanding of disease progression and the tissue destruction that facilitates the spread of the bacteria to the circulation, hence the spread to distant tissues. The identification of increased risk by low levels of antibodies of TF for CVD mortality and bladder cancer and TD for bladder and colon cancer in the Oslo II study, Norway, opens the “window” of case identification (8, 9).

## Short descriptions of the most relevant bacteria

### The red complex

*Tannerella forsythia* is a Gram-negative anaerobe bacterium involved in the pathogenesis of periodontitis (17). It is relatively poorly studied due to its slow growth in the laboratory. TF can break down collagen I and III. Collagen is a major constituent of the periodontium with collagen type 1 accounting for 80%–85% of the gingiva and 90% of the alveolar bone. The ability of TF to break down collagen is important in tissue-destructive infections. In the outer membrane of TF are 221 proteins identified but their function is not yet clarified for all.

*Porphyromons gingivalis* is a Gram-negative oral anaerobe. Several of its metabolic properties have been mapped, and it is identified as an etiologic agent and the cause of severe adult periodontitis (18). In the biofilm surrounding the teeth, it adheres quickly to the epithelial cells in the periodontium. It causes deregulation of innate immune and inflammatory responses (18). The lipopolysaccharide of PG can activate host inflammatory and innate defense responses. It stimulates osteoclast production that initiates alveolar bone resorption.

*Treponema denticola* is an oral anaerobe bacteria associated with chronic periodontitis (19). It is an invasive spirochaete. Its major virulence factor is chymotrypsin-like proteinase (Td-CTLP) and this has been studied in tumor tissue of orodigestive tumors. Td-CTLP was found to convert pro-MMP-8 and -9 into their active forms. In addition, Td-CTLP was able to degrade the proteinase inhibitors TIaMP-1, TIMP-2, and α-1-antichymotrypsin, as well as complement C1q. Nieminen et al. concluded that because of its observed presence within tumors and its known regulatory activity on proteins, it is critical for the regulation of the tumor microenvironment and inflammation. The Td-CTLP may contribute to orodigestive carcinogenesis.

### Some other relevant oral bacteria

*Fusobacterium nucleatum* (FN) is a Gram-negative fusiform bacteria that is abundant in the gut and the mouth, especially in dental plaque (3). There are several subspecies. They are proteolytic and form cytotoxic end products. It belongs to the orange complex (3).

*Aggregatibacter actinomycetemcomitans* (AA) is abundant in the oral cavity, and is present in subgingival plaque in aggressive periodontitis. It is a facultative anaerobe coccoid Gram-negative rod (3). It has 10 subtypes and has different properties, one of which is to kill leucocytes and monocytes. It also forms immunoglobulin proteases and collagenase.

*Prevotella intermedia* (PI) is also anaerobic and is a slow-growing Gram-negative coccoid rod (3). It produces lipase when involved in bacterial invasion of the periodontium. Of the 30 subspecies, some are members of the orange complex. It is characterized by its proteolytic metabolism.

In summary, these oral bacteria possess collective properties that contribute to their survival within the oral cavity depending on the degree of the anaerobe condition. The breakdown of periodontal tissue and alveolar bone tissue provides material for their joint survival. These bacteria have been identified in different cancer tissues and cardiovascular structures, and their ability to live in anaerobe tissue is considered important but their role in causing systemic diseases is not fully established.

## Cardiovascular diseases and oral health

### Identification of anaerobe bacteria in CVD sites

Anaerobe bacteria have been identified in human tissue in several CVD sites. These include coronary artery walls specifically within the cells of the walls and within atherosclerotic plaque. Their invasive properties are shown by bacterial DNA in stenosed/calcified heart valves, aneurysms, and infective endocarditis. In addition, DNA from other bacteria have been identified in calcified heart valves (1, 2, 13). There are three major causal mechanisms in which one considers oral bacteriology to be involved: atherosclerosis; thrombosis formation; and immunology (2). Several systematic reviews show associations with myocardial infarction, stroke, peripheral arterial disease, and infective endocarditis. Oral health and CVD have been recorded, observed, and measured differently in many studies and summarized in systematic reviews. CVD is a multifactorial disease and the results in studies have been adjusted for common confounders. The most important are smoking, body mass index, social status/education level, elevated blood pressure, and high cholesterol level.

### Myocardial infarction

In a review of the literature on the association between CVD and oral disease, a selection of studies shows (1, 2) that myocardial infarction/ischemic heart disease has been investigated by reported tooth loss, total dental index, Russell's periodontal index, alveolar bone level, mild gingivitis, severe gingivitis, or periodontitis (20). Six studies showed a significantly increased risk using these oral health parameters. They also reported three non-significant studies. These studies measured the risk as reported periodontitis or Russell's periodontal index. Standardized measurements give the most exact results, such as periodontal pocket depth, number of teeth, and loss of alveolar bone. The outcomes were incidence or mortality by CVD, and coronary heart disease (CHD). Almeida et al. published a systematic review of studies on oral Gram-negative bacteria and their association to atherosclerosis (21). Of 2,138 studies identified, 4 cohort studies were included. Comparing groups with or without periodontitis, they found an association between atherosclerosis and periodontitis to be associated with inflammatory CRP and IL-6. Bacterial DNA has been identified in stenosed heart valves and aneurysms of the descending aorta (22, 23). Okuda et al. published a review of studies on the process by which oral anaerobe bacteria enter the arterial wall (24). They include electron micrographs that show bacteria present in the arterial wall. They refer to several studies and cover different mechanisms that have been observed in a variety of scientific studies.

Larvin et al. reported an extensive systematic review including 30 studies in 2021 (25). They found that the risk of incident CVD was higher in individuals with periodontal disease (PD) than in those without [relative risk (RR) = 1.20, 95% CI 1.14–1.26]. The risk of stroke was the highest of the CVD diagnoses (RR = 1.24, 95% CI 1.12–1.38).

Joshi et al. examined coronary atheroma and the prevalence of oral bacteria (26). They identified 14 studies and found PG and AA to be the most prevalent, PG more so than AA. Other recognized periodontal bacteria were *Pseudomonas fluorescens*, *Streptococcus* spp., and *Chlamydia pneumoniae*. Identifying oral bacteria in the atheroma is a clear indication of periodontal disease as part of the causal picture in CVD. The association between apical periodontitis and CVD is mixed. Berlin-Broner et al. found 19 eligible studies with varying results (27). They varied in study design. Thirteen were positive, five were not, and one was negative in linking apical periodontitis to CVD.

### Stroke, atrial fibrillation, and peripheral arterial disease

Oral health has been studied for the risk of stroke by oral health status, number of extractions, remaining teeth, alveolar bone loss, gingivitis, and periodontitis. An early prospective study was performed by the National Health and Nutrition Examination Study (NHANES) (28). Nearly 10,000 individuals aged 25–74 years participated. They found periodontitis to be predictive of total cerebrovascular events (HR = 1.66, 95% CI 1.15–2.39) and non-hemorrhagic stroke (HR = 2.11, 95% CI 1.30–3.42). In the large Health Professional Follow-up Study (HPFS), they found that men with <25 teeth at baseline had an elevated risk compared to men with more teeth (HR = 1.57, 95% CI 1.24–1.98) (29). The association with ischemic stroke had an HR of 1.33 (95% CI 1.03–1.71). Other studies confirm these associations (1). In the Oslo II study on elderly men, analyses at 12.5 years of follow-up showed an increased risk of cerebral infarction (HR = 2.92, 95% CI 1.24–6.89) for >10 teeth extractions due to infections, but not for cerebral hemorrhage or unspecified stroke (30).

Atrial fibrillation was studied in the dental Atherosclerosis Risk in Communities study (31). Severe PD increased the risk for atrial fibrillation (HR = 1.31, 95% CI 1.02–1.62).

Peripheral arterial disease has also been studied, and German, Chinese, and American researchers have found significant associations between oral health and peripheral arterial disease (32–35).

### Infective endocarditis

The main focus concerning infective endocarditis for dentists is prophylaxis. Guidelines differ in terms of using antibiotics prophylactically. Infective endocarditis can result in heart valve disease and thrombus formation, followed by a cerebral infarct. Antibiotic-resistant bacteria have become a great problem in the treatment of patients. After the introduction of a new antibiotic, the bacteria develop “strategies” to survive and therefore narrow our treatment options.

### Serum analyses

In the Oslo II study from the year 2000, men with the lowest quartile of antibodies to *Tannerella forsythia* had an increased risk of CVD mortality in men with a history of MI compared to men with no history of MI during 12.5 years of follow-up (HR = 1.82, 95% CI 1.12, 2.94) (6). From this study, we have an analysis of the blood microbiome that was mapped on the genera level. Men dying from CVD were compared to a random sample of men selected from the study cohort. Bacterial DNA was found in 372 (82%) of the blood samples and 78 genera were identified. This reflects the prevalence of bacterial DNA in the blood and the great diversity. The following three genera were significant predictors after Bonferroni adjustment of the analyses: *Kocuria* (adjusted HR = 8.50, 95% CI 4.05–17.84); *Enhydrobacter* (HR = 3.30, 95% CI 2.01–5.57); and *Paracoccus* (HR = 0.29, 95% CI 0.15–0.57) (36).

Fortunately, a wide scope of risk factors and disease endpoints have been used in these studies. This allows for a broad view that gives credit to the common findings of increased risk for CVD related to poor oral health.

## Cancer and oral health

Certain bacteria and viruses play a causal role in the etiology of specific cancers. The best known are *Heliobacter pylori* (*H. pylori*), which causes gastric cancer, and *human papillomavirus* (HPV), causing cervical, oropharyngeal, vagina, anus, and penis cancers (37, 38). A vaccine has been developed against HPV. Oral health with a focus on periodontal health has been studied for its positive association with head and neck, lung, breast, stomach and esophageal, pancreatic, colorectal, bladder, prostate, corpus uteri, hematopoietic, and lymphatic cancers in specific systematic reviews and single studies (1).

Smoking has been a major risk factor for oral squamous cell carcinoma (OSCC), but smoking cessation initiatives have reduced its incidence. Treatment is difficult although these cancers are often discovered early in their development. The oral cavity is also exposed to osteoporosis as a side effect of radiation and drug treatment, especially for breast cancer. Cancer of the lip and oral cavity is the 13th most common cancer in the world and the third most common in some Asian Pacific countries according to the World Health Organization (https://w.w.w.who.int/news-room/fact-sheets/detail/oral-health). Oral cancer here includes cancers of the lip, other parts of the mouth, and the oropharynx, is more common in men, and it varies according to socioeconomic factors.

### Three large systematic reviews of prospective studies

Large systematic reviews have been written covering studies from different parts of the world, including Europe, Asia, and North America. Xiao et al. evaluated whether periodontal infection was associated with the incidence and prognosis of cancer (39). Their literature search included 39 studies: 3 from Europe, 16 from Asia, and 19 from North America. They reported that periodontitis increased the incidence of cancer (HR = 2.18, 95% CI 1.24–3.84) and poor overall survival (HR = 1.75, 95% CI 1.40–2.20). In addition, they found PG infection to be associated with an odds ratio (OR) of 2.16 (95% CI 1.34–3.47) in 10 studies. *Prevotella intermedia* and TD were also associated, but not TF, AA, or FN. Sayehmiri et al. did not find an increased risk with PD (40).

Michaud et al. finally selected 46 studies on the risk of self-reported periodontitis and different cancers based on 52 studies from previous reviews and 217 reviews published between 2011 and 2016 (41). They presented meta-analyses for cancer of the lung and pancreas, and pooled estimates in a long-term follow-up. The relative risk for lung cancer was 1.33 (95% CI 1.19–1.49) and 1.2 (95% CI 1.0–2.4) for pancreatic cancer.

Corbella et al. included 6 studies of 10 papers out of a total of 490 papers (42). These studies included registrations for several different cancers: The Swedish Twin Registry: total cancer, digestive tract, colorectal, pancreas, stomach, bladder, prostate, breast corpus uteri, and lungs; a study from Boston, USA: oral, pharynx, larynx; from the Women's Health Initiative Observational Study, USA: lungs and breast; from the Male Health Professional Study, USA: pancreas and total cancer; from the Nurses Study, USA: colorectal; and from the Carolina Head and Neck Cancer Epidemiology study, USA: head and neck squamous cell carcinoma.

In summary, all these studies and several more explore the possible association between oral health and cancer in different organs of the body.

## Immunology and microbiology in cancer

Al-Hebshi et al. have described the microbiome of oral squamous cell cancer (OSCC). They identified four bacteria that were involved: PG, FN, *Neisseria* spp., and *Streptococcus* spp. (43). One or more of these were involved in the following biological functions: production of carcinogen metabolites; inhibition of apoptosis; activation of cell proliferation; activation of cellular invasion; or induction of chronic inflammation. They concluded from their findings that no one microbial species is involved in the etiology of OSCC; instead, they contribute to tumor progression by sustaining chronic inflammation. FN produces butyric acid, which is a source of energy for anti-inflammatory cells. FN plays a part in the activation of the immune system (44). The prevalence of FN increases in periodontal disease and the severity of the disease through inflammation and in the depth of periodontal pockets. FN exhibits several functions in several diseases, but concerning colorectal cancer, it is believed that outer membrane proteins take part in the cancer developing process.

Minarovits et al. examined how the anaerobes of the oral biofilm are found intratumoral in OSCC (45). In the oral cavity, the biofilm adheres to the oral mucosa including any OSCC developments. The identification of anaerobes within tumor tissue is of great interest when studying malignant growth. These researchers found nine common bacterial species in both OSCC samples and control samples. In OSCC samples, *Micrococcus luteus* (an aerobe), *Prevotella melaninogenica*, *Exiguobacterium oxidotolerans*, *Stapholococcus auresu*, *Veillonella parvula*, and *Fusobacterium naviforme* were also identified.

Another approach was taken in the Oslo II study analyzing for antibodies to the red complex bacteria and AA (9). The risk of cancer was found after 17.5 years of follow-up. *Tannerella forsythia* predicted bladder cancer and *Treponema denticola* predicted both bladder cancer and colon cancer. The risk was reported inversely by lowest quartile value versus fourth quartile; TF inversely predicts bladder cancer (*n* = 22; HR = 1.71, 95% CI 1.12–2.61), and TD inversely predicts colon cancer (*n* = 26; HR = 1.52, 95% CI 1.06–2.19) and bladder cancer (*n* = 22; HR = 1.60, 95% CI 1.05–2.43). The results indicate that a lowered immunologic response is important in the development of cancer and a characteristic of individuals at increased risk of cancer. The recent pandemic taught us that viruses can cause serious disease. We all needed a vaccine to curb the pandemic because no one had antibodies to this new variant. Oral bacteria are not rare, and most people have sufficient antibodies to curb the systemic spread of these bacteria that are so prevalent. There are, however, individual differences.

## Problems and possibilities according to study design

Different study designs provide different opportunities for establishing the level of evidence. In planning or assessing scientific studies, the following issues should be considered.

### Association or causality

In studying the effect of a measure, the randomized controlled study (RCT) is considered an optimal choice. Looking at the cause of a disease, the prospective cohort that starts as a cross-sectional study is important. The prospective judgment of the treatment effect or change in risk factors for a disease provides important temporal evidence for study outcomes. If it is a rare condition, then case-control studies may be the first option to gain more knowledge. Case series have the importance of mapping, e.g., treatment effects that can generate new hypotheses to be tested. If systematic reviews are available, they provide summary measures across several studies. Well-conducted studies provide important and valuable information.

### Validity of a study

Carrying out research is a demanding process and must be assessed and described carefully. In approaching association and causality, we need to ascertain that the execution of the study and its results are valid. Important issues are confounding, selection bias, information bias, misclassification, and surveillance bias (46). New hypotheses as oral anaerobe bacteria being associated or causal to both CVD and cancer need to be documented using an epidemiologic approach. Understanding Hill's criteria of causality gives an approach to what type of knowledge is required and why (47, 48). His nine criteria were strength, consistency, specificity, temporality, biological gradient, plausibility, coherence, experiment, and analogy.

### Microbiology, serum, tissue samples

In studying antibodies, the theories of microbiology and immunology are necessary to understand this issue. Analyses of serum samples and tissue samples can provide important information about the function and dysfunction of metabolic processes. In addition, registries (local or national) of disease-specific occurrence, incidence, and/or mortality can provide important knowledge of disease development preferably compared to healthy individuals.

## What scientific literature supports or contravenes this hypothesis

Very few papers report no significant association between oral health and systemic disease, which may be an effect of publication bias. However, risk estimates are stable and significant in most studies that have been published. The studies are often summarized in systematic reviews and come from different populations and countries around the globe (1, 2). This gives strength to the evidence.

Research results have been presented with a strong focus on different aspects of oral infections to further our understanding of this relationship. Many different oral health and microbiologic measures have been used to map this association. The focus has been on the major cardiovascular diseases and cancer diagnoses. Registry data have often been used to ascertain outcomes. The oral risk has been recorded either by self-reported information, clinical information, X-ray images, or serological measurements. The level of precision of these exposure variables has been important to maintain throughout the study period. Equivalently stringent analyses and reporting have to be carried out professionally to trust the results when we are moving into new research areas considering oral anaerobe bacteria as a common bacteriological/immunological causal pathway for CVD and cancer. In cancer, the oral microbiome has predominantly been studied in OSCC (48). Oral anaerobe bacteria have been identified in several other forms of cancer (1, 49).

Much attention has come from alcohol and its prevention against MI at low daily levels. However, alcohol also kills bacteria, and while swallowing alcohol, it passes the habitat of the anaerobe bacteria at the back of the tongue and throat. It was observed that a regular level of alcohol drinking 4–6 times per week was associated with fewer tooth extractions and a non-significant risk of total mortality versus less alcohol drinking 1–2 times per week (15). Another published analysis showed that increasing numbers of tooth extractions due to infections were associated with increased total mortality (50).

In total, there seems to be a relation between the oral anaerobe bacteria TF and CVD independent of other well-known risk factors. Our additional prospective finding of an association to bladder and colon cancer by low antibody levels to TF and TD is very interesting. A lot of research, some reported here, shows the involvement of oral anaerobe bacteria in both CVD and certain forms of cancer. In the studies reported by Psaty, there were no reported microbiologic measurements of bacteria but diagnosed cases. The results of the Oslo II study are suggestive of a reduced immunologic response to oral anaerobe bacteria playing a causal role in these diseases in the same individuals. The biological mechanisms are so far unknown as the bacteria possess numerous properties that may act together enabling the bacteria to invade and/or survive in tissues distant to the oral cavity. However, the current state of knowledge is not sufficient to allow any clinical measures.

## Conclusion

This research suggests an association between oral anaerobe bacteria and systemic diseases regulated by the immune system through antibody production as shown for CVD and specific forms of cancer. Further research is needed to explore the biological mechanisms that determine these pathways. Optimal oral health has always been important by itself, and this research underscores its significance with regard to systemic diseases.
